# Pericostal tuberculosis in a patient with systemic sclerosis:The relationship of two rare diseases

**DOI:** 10.1002/ccr3.4563

**Published:** 2021-08-30

**Authors:** Naoho Takizawa, Tetsushi Mizutani, Yoshiro Fujita

**Affiliations:** ^1^ Department of Rheumatology Chubu Rosai Hospital Nagoya Japan; ^2^ Department of Surgery Chubu Rosai Hospital Nagoya Japan

**Keywords:** *Mycobacterium tuberculosis*, scleroderma, systemic sclerosis, tuberculosis

## Abstract

Regardless of immunosuppressant use, physicians should be aware of pulmonary and extra‐pulmonary tuberculosis in patients with autoimmune disease including systemic sclerosis, especially if they follow unusual clinical courses.

## Graphical abstract

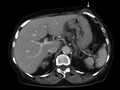

## 1 INTRODUCTION

Autoimmune diseases including systemic sclerosis (SSc) increase risk of developing TB. Pericostal tuberculosis (TB) is a rare presentation of skeletal TB. This case report describes pericostal TB in a SSc patient and emphasizes significance of suspecting pulmonary and extra‐pulmonary TB when patients with autoimmune disease follow atypical clinical courses.

**FIGURE 1:**
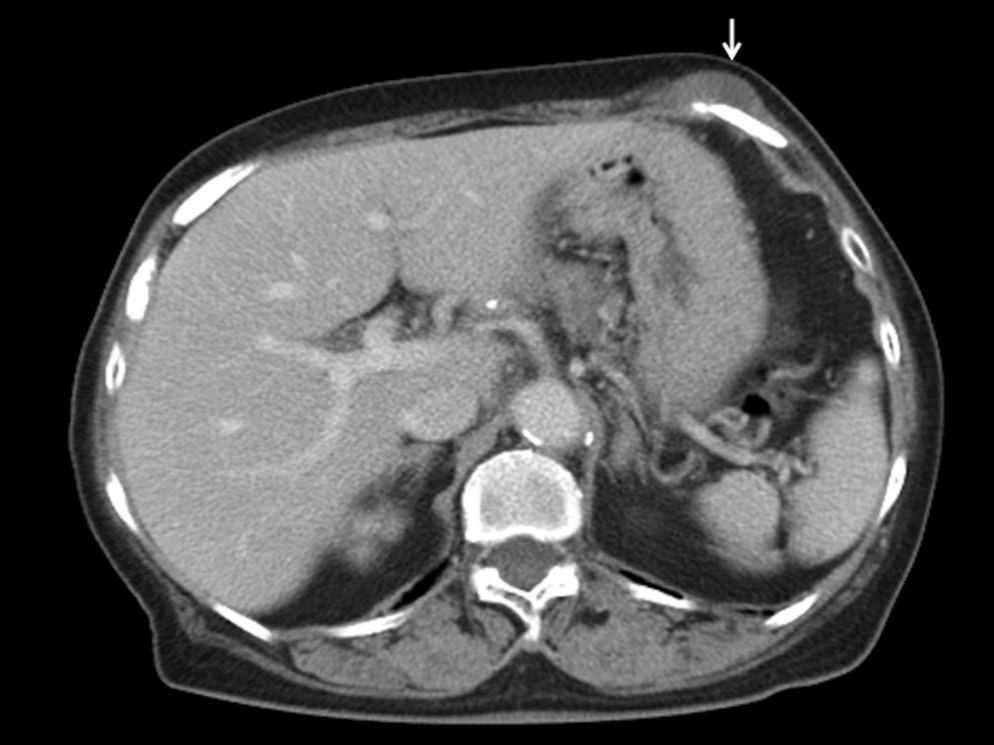
Abdominal contrast‐enhanced CT. White arrow showed pericostal mass with central hypoattenuation

An 83‐year‐old woman presented with a left pericostal painful mass which she had had for a year. She was diagnosed with systemic sclerosis (SSc) by skin thickening and a positive result of anti‐centromere antibody at age 75, however, she did not take any immunosuppressants. An abdominal CT showed a pericostal mass (Figure 1). We performed a needle biopsy, and the result was negative for Ziehl‐Neelsen stain, but both the PCR and culture of tuberculosis (TB) from the drain were positive. She was diagnosed with pericostal TB, and we started a combination treatment with isoniazid, rifampicin, pyrazinamide, and ethambutol. Her chest pain resolved quickly and a repeat biopsy culture was done one month after initiation of treatment, and the result was negative. Pericostal TB is a rare presentation of skeletal TB and is thought to be caused by an extension of a TB infection of the intercostal lymph nodes.^1^ It has been reported that the risk of TB in SSc patients is 2.8 times higher than those in the general population.^2^ The increased risk of developing TB in patients with autoimmune disorders may be due to an immune abnormality itself or immunosuppressants.^2^ Regardless of immunosuppressant use, physicians should be aware of TB in SSc patients.

## CONFLICT OF INTEREST

None declared.

## AUTHOR CONTRIBUTIONS

Naoho Takizawa: wrote the initial draft, reviewed the literature, revised manuscript, and approved the final version. Tetsushi Mizutani: reviewed the literature and revised the manuscript. Yoshiro Fujita: reviewed the literature, revised the manuscript, and approved the final version.

## ETHICAL STATEMENT

Written informed consent was obtained from the patient who participated in this study. This case report did not receive any funding. Authors have access to all source data for this case report.

## Data Availability

The data that support the findings of this study are available on request from the corresponding author. The data are not publicly available due to privacy or ethical restrictions.
